# Mid-ventricular obstruction in a patient with hypertrophic cardiomyopathy

**DOI:** 10.31744/einstein_journal/2022AI6672

**Published:** 2022-04-13

**Authors:** Kevin Rafael De Paula Morales, Cristhian Vicente Espinoza Romero, Williams Roberto Lata Guacho, David Alejandro Salazar Jaya, Eduardo Kaiser Ururahy Nunes Fonseca

**Affiliations:** 1 Hospital das Clínicas Faculdade de Medicina Universidade de São Paulo São Paulo SP Brazil Instituto do Coração, Hospital das Clínicas, Faculdade de Medicina, Universidade de São Paulo, São Paulo, SP, Brazil.

**Figure 1:**
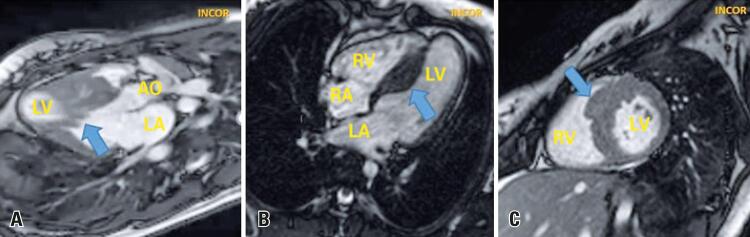
Steady-state free precession sequence cardiovascular magnetic resonance in systole and diastole. (A) Longitudinal plan of three chambers in systole, showing asymmetrical myocardial hypertrophy, with septal predominance and systolic anterior movement of the anteroseptal mitral valve muscle (arrow); (B) Coronal plan of four chambers, with septal hypertrophy (arrow); (C) Middle segment axial plan, which showed septal hypertrophy (arrow)

LV: left ventricle; AO: aorta; LA: left atrium; RV: right ventricle; RA: right atrium.

**Figure 2:**
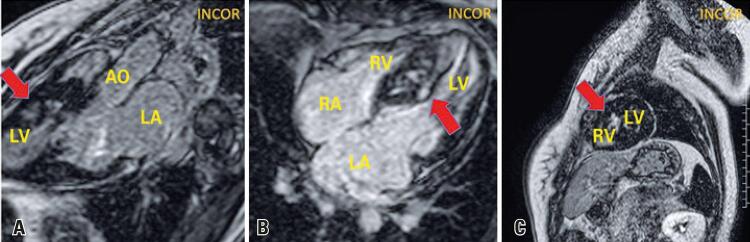
Tissue characterization by late enhancement sequence. (A) Longitudinal plan of three layers showing heterogeneous ischemic fibrosis in the middle segment of the septum (arrow); (B) Coronal plan of four chambers, showing hypertrophy and non-transmural fibrosis in the septum (arrow); (C) Axial plan of the middle segment, with septal myocardial fibrosis (arrow)

AO: aorta; LA: left atrium; LV: left ventricle; RV: right ventricle; RA: right atrium.

An 18-year-old female patient with hypertrophic cardiomyopathy previously asymptomatic, diagnosed in family screening, who presented progressive dyspnea even upon mild exertion two months before. She was submitted to cardiac magnetic resonance (Figures 1 and 2), which showed asymmetrical myocardial hypertrophy with mid-ventricular septal predominance. The most common form of obstruction of the left ventricle outflow tract in hypertrophic cardiomyopathy is subaortic obstruction,^(1)^ which generally results from left ventricle outflow tract narrowing by septal hypertrophy and systolic anterior motion of the mitral valve anterior cuspid.^(2)^

Another more rare obstructive mechanism is that resulting from impedance to flow in the middle of the left ventricular cavity, called mid-ventricular obstruction, a distinct phenotype of hypertrophic cardiomyopathy, occurring in approximately 10% of patients.^(3)^ It is essentially caused by two mechanisms: the impact of the hypertrophied septum in the left ventricle free wall, generally with interposition of the hypertrophied papillary muscle,^(3)^ and anomalous insertion of the hypertrophied anterolateral papillary muscle directly in an anterior elongated mitral leaflet.^(4)^

The diagnosis of mid-ventricular obstruction is considered when there is a mid-ventricular gradient estimated at 30mmHg. Obliteration is caused by marked septal hypertrophy, resulting in contact with the hypercontractile left ventricle free wall, and not by systolic anterior motion of the mitral valve anterior leaflet.^(5)^

Patients with mid-ventricular obstruction tend to present many symptoms - dyspnea is the most common, and have increased risk of progressive heart failure and death (sudden death and arrhythmic events), according to studies in this population.^(3,5,6)^ Moreover, the formation of apical aneurysms is more frequent in this subtype of hypertrophic cardiomyopathy.^(5)^

The initial treatment of this condition is usually conservative. The interventions are reserved for cases of persistent symptoms after initiating drug therapy.^(7)^

After assessing the images, discussing the case, and establishing the obstruction mechanism, she initiated on beta blocker, with appropriate initial response. It was decided to carry on drug therapy, with strict clinical follow-up, due to the risk of complications associated with the disease.
